# ADAMTS1 protease is required for a balanced immune cell repertoire and tumour inflammatory response

**DOI:** 10.1038/s41598-018-31288-7

**Published:** 2018-08-30

**Authors:** Francisco Javier Rodríguez-Baena, Silvia Redondo-García, Carlos Peris-Torres, Estefanía Martino-Echarri, Rubén Fernández-Rodríguez, María del Carmen Plaza-Calonge, Per Anderson, Juan Carlos Rodríguez-Manzaneque

**Affiliations:** GENYO. Centre for Genomics and Oncological Research: Pfizer/Universidad de Granada/Junta de Andalucía, Avda. de la Ilustración, 114, Granada, 18016 Spain

## Abstract

Recent advances have emphasized the relevance of studying the extracellular microenvironment given its main contribution to tissue homeostasis and disease. Within this complex scenario, we have studied the extracellular protease ADAMTS1 (*a disintegrin and metalloprotease with thrombospondin motif 1*), implicated in vascularization and development, with reported anti- and pro-tumorigenic activities. In this work we performed a detailed study of the vasculature and substrates in adult organs of wild type and *Adamts1*-deficient mice. In addition to the expected alterations of organs like kidney, heart and aorta, we found that the lack of ADAMTS1 differently affects lymphocyte and myeloid populations in the spleen and bone marrow. The study of the substrate versican also revealed its alteration in the absence of the protease. With such premises, we challenged our mice with subcutaneous B16F1 syngeneic tumours and closely evaluated the immune repertoire in the tumours but also in the distant spleen and bone marrow. Our results confirmed a pro-inflammatory landscape in the absence of ADAMTS1, correlating with tumour blockade, supporting its novel role as a modulator of the immune cell response.

## Introduction

The extracellular matrix (ECM) is a basic constituent of all tissues and organs, serving as a highly dynamic structure in constant interaction with surrounding cells. The ECM undergoes a continuous remodelling according to the characteristics and functionality of every specific niche, entailing the participation of extracellular matrix proteases^1^. The study of this multifaceted scenario stimulated initiatives like the Matrisome^2^ and the Degradome^3^, aiming to characterize the components of the ECM and their functional interactions. Certainly, the impact of an altered proteolysis in the microenvironment keeps being updated by studies using genetically modified animal models and in particular from cancer-related reports^4^. However, there is still much work to be done in order to fully decipher the complexity around ECM dynamics. In this context, the ADAMTS (*a disintegrin and metalloprotease with thrombospondin motif*) family represents a group of extracellular proteases belonging to the zinc-dependent metzincin superfamily^5^, which actions in the extracellular microenvironment are still being elucidated. Since the first discovery of ADAMTS1^6^, various reports have described its angiostatic and tumour blocking properties although its definitive contribution to vascularization is still debated^7^. In fact, other studies have proposed a pro-metastatic and tumorigenic activity of ADAMTS1^8,9^. *Adamts1* knockout (Ats1-KO) mice exhibit reduced body weight, kidney malformation, and impaired female fertility^10,11^. Additional reports suggested a contribution of this protease in myocardial morphogenesis^12^ and its deficiency has also been correlated with aortic aneurysms^13^. Finally, the catalytic activity of ADAMTS1 has been reported on various proteoglycans^14–16^, and further extracellular components^17–19^.

The aim of the current study was to take a closer look on the adult organs in wild type and *Adamts1* knockout mice, mainly evaluating the status of their vasculature and specific ADAMTS1 substrates with recognized relevance in the microenvironment, such as nidogens and versican, In one side, our work showed variations of vascular markers and substrates in adult organs that have been correlated with previous phenotypic annotations, corroborating the activity of ADAMTS1 on the vasculature. Furthermore, and unexpectedly, the spleen and bone marrow appeared affected by the lack of ADAMTS1. These findings warrant a thoughtful review of the involvement of this protease and further members of the ADAMTS family in inflammatory and immune responses. Indeed, the intimate relationship of the extracellular microenvironment with a immunomodulatory response during tumour progression has been strongly supported by later advances^4,20^. Alongside, our investigation includes B16F1 tumour-bearing Ats1-KO mice, allowing the identification of previously unknown activities of ADAMTS1 in immune organs. These results warrant new investigations that can improve our understanding of tumour-immune interplay and immunomodulation.

## Results

### Evaluation of substrates and vasculature in adult organs of wild type and Ats1-KO mice

**Figure 1:**
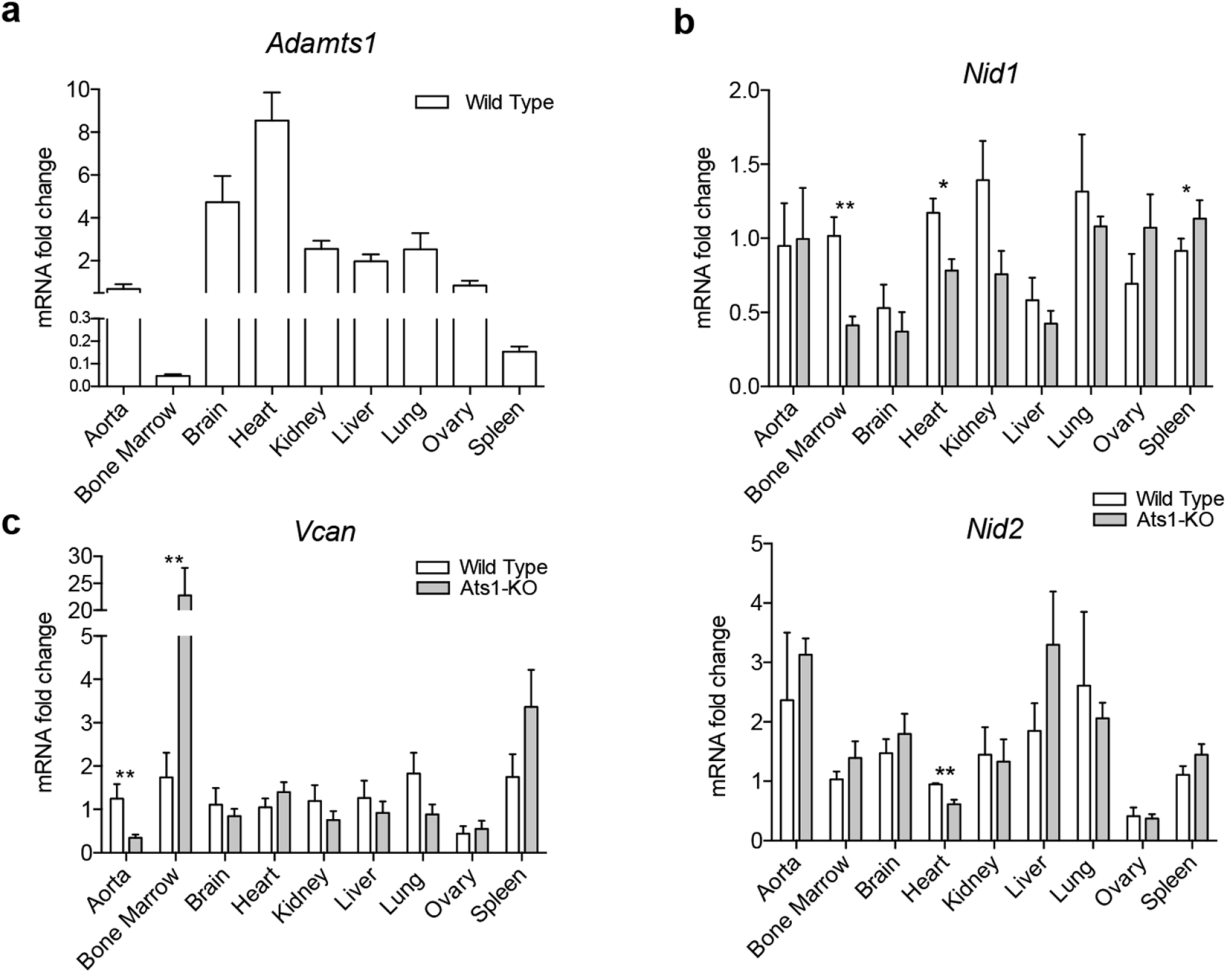
Gene expression of *Adamts1* and its substrates nidogens and versican in WT and Ats1-KO mice. **(a)** Graph representing mRNA fold change expression of *Adamts1* in WT organs (n = 5), relative to aorta. **(b)** Graphs representing relative mRNA fold change expression of substrates *Nid1* and *Nid2* in organs from WT (n = 5) and Ats1-KO (n = 5) mice. **(c)** Graphs representing relative mRNA fold change expression of substrate *Vcan* in organs from WT (n = 5) and Ats1-KO (n = 5) mice. All results in graphs are expressed as the median with s.e.m. and statistical significance (*p < 0.05; **p < 0.01).

To obtain a comprehensive view of the biological actions of the protease ADAMTS1 we performed a gene expression study in adult mouse organs. First, we evaluated the expression of *Adamts1* in aorta, bone marrow (BM), brain, heart, kidney, liver, lung, ovary and spleen, finding the highest levels in heart and brain, and the lowest expression in BM and spleen (Fig. 1a). We also analysed the expression of its substrates nidogens (*Nid1* and *Nid2*)^17^, and versican (*Vcan*)^16^, with a recognized role in specialized ECMs. To our surprise, significant differences were observed between wild type (WT) and Ats1-KO mice in two immune-related organs, the BM and spleen. In the case of *Nid1* we found an opposite alteration, being downregulated in BM of Ats1-KO mice, but upregulated in the spleen of these animals (Fig. 1b). For *Vcan*, we observed a strong induction in the BM of Ats1-KO mice and a similar tendency in spleen but without statistical significance (Fig. 1c). In addition, hearts of Ats1-KO mice showed a relevant downregulation of both *Nid1* and *Nid2* (Fig. 1b), and their aortas presented lower levels of *Vcan* (Fig. 1c), suggesting an association with previous references on myocardial morphogenesis and development of the aorta, respectively^12,13^. According to the recognized actions of ADAMTS1 on the vasculature, we extended this analysis to common vasculature-related markers, such as *Pecam1* (CD31), widely identified at endothelial cell-cell contacts^21^, *Cspg4* (NG2), recognizing mural supporting cells of neovasculature^22^, and *Acta2* (alpha 2 smooth muscle actin), the main marker of vascular smooth muscle cells^23^ (Supplementary Fig. 1a). With the exception of kidney and aorta, the analysis of these genes did not show major alterations. We detected a downregulation of *Cspg4* in kidneys of Ats1-KO mice, probably associated with the already reported deficient maturity of this organ^11^. Regarding adult aortas, the decreased levels of *Pecam1* and *Acta2* in Ats1-KO mice again correlated with the described deficiency of ADAMTS1 during heritable aortic aneurysms as the Marfan syndrome^13^. Given the mentioned alterations of substrates in the spleen and BM, we also visualized and quantified the vasculature of these organs by immunofluorescence (Supplementary Fig. 1b,c).

### The study of spleen and bone marrow of Ats1-KO mice reveals the alteration of immune populations and versican

**Figure 2:**
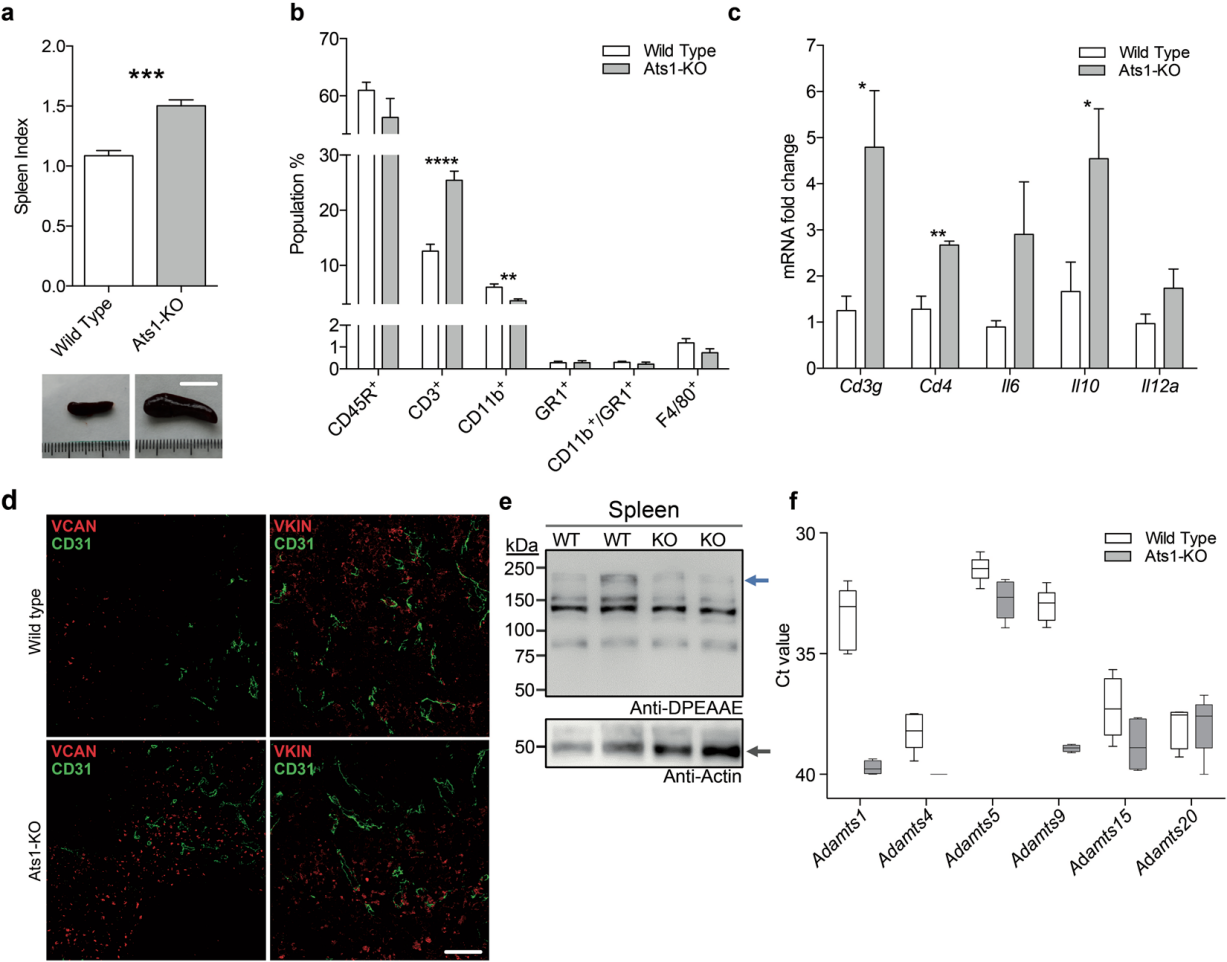
Characterization of spleens from WT and Ats1-KO mice. **(a)** Graph representing the spleen index (as indicated in the Methods section) of healthy WT (n = 6) and Ats1-KO (n = 5) mice to assess splenomegaly. Inset below include representative images of spleens from WT and Ats1-KO mice (white scale bar = 1 cm). **(b)** Graph representing flow cytometry data, as percentage of positive cells of the following populations: CD45R^+^, CD3^+^, CD11b^+^, GR1^+^, CD11b^+^/GR1^+^, and F4/80^+^, found in spleens of healthy WT (n = 5) and Ats1-KO (n = 5) mice. **(c)** Graph representing the mRNA fold change expression of *Cd3g*, *Cd4*, *Il6*, *Il10* and *Il12a* genes in spleens of healthy WT (n = 5) and Ats1-KO (n = 5) mice, being all values relative to WT. **(d)** Representative images of spleen sections from WT and Ats1-KO mice, showing VCAN and VKIN (red) (left and right column, respectively), and CD31 (green) immunofluorescence staining. Images correspond to a 63x magnification (white scale bar = 20 μm). **(e)** Western blot analysis with anti-DPEAAE antibody for ADAMTS-cleaved versican in spleen protein extracts from WT and Ats1-KO mice. Blue arrow indicates a predicted versican (V0) fragment (around 220 kDa). Bottom panel includes actin staining (grey arrow). Full-length blots are presented in Supplementary Figure 4. **(f)** Graph representing the absolute Ct value for mRNA expression of *Adamts1*, *4*, *5*, *9*, *15*, *and 20* genes in spleens of healthy WT (n = 5) and Ats1-KO (n = 4) mice (values higher than 37–38 are considered very low or absent). For all the graphs, results are shown as the median with s.e.m. and statistical significance (*p < 0.05; **p < 0.01; ***p < 0.001; ****p < 0.0001).

We next performed a deeper study of the spleen and BM. For the spleen, our analysis revealed a consistent and significant splenomegaly in Ats1-KO mice in comparison with WT mice (Fig. 2a). The evaluation of hematoxilin and eosin (H&E) stained sections of spleens did not display significant alterations between WT and Ats1-KO samples (Supplementary Fig. 2a). We then performed carboxyfluorescein diacetate succinimidyl ester (CFSE) proliferation assays on freshly isolated splenocytes to test the *ex-vivo* proliferative activity of T-cell populations^24^. However, neither this assay disclosed significant differences (Supplementary Fig. 2b). Finally, we used flow cytometry (as detailed in Methods and Supplementary Fig. 3) to analyse any possible intrinsic changes in the main immune cell populations of this organ. The highly represented population of CD45R^+^ cells was not affected. However, we detected a remarkable increase in CD3^+^ cells (T cells) in spleens of Ats1-KO mice (Fig. 2b). In addition, myeloid CD11b^+^ cells decreased in Ats1-KO mice, while other populations, such as GR1^+^, CD11b^+^/GR1^+^, and F4/80^+^, were not affected (Fig. 2b). The indicated increase in T cells, that suggests a pro-inflammatory scenario in spleens of Ats1-KO animals, was also supported by gene expression analyses of relevant genes like *Cd3* (*Cd3g*)^25^, together with *Cd4*^26^, *Il6*, *Il10* and *Il12a*^27–29^ (Fig. 2c).

We next performed a deeper study of VCAN. Indeed, immunofluorescence analyses confirmed an increased deposition of VCAN in spleens of Ats1-KO mice in comparison with WT (Fig. 2d). However the evaluation of the processed form of VCAN, named versikine (VKIN)^16^, displayed a similar deposition between WT and Ats1-KO spleens (Fig. 2d). Western blot analyses also showed that proteolysis of VCAN, although partially affected by the absence of endogenous *Adamts1*, still occurred at a relevant rate (Fig. 2e), confirming the participation of additional proteases. We evaluated the gene expression of other ADAMTSs with versicanase activity, showing a significant level of *Adamts5* in this organ (Fig. 2f).

**Figure 3:**
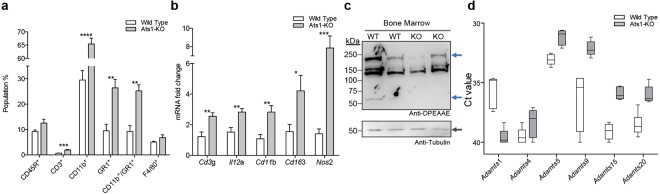
Characterization of bone marrow from WT and Ats1-KO mice. **(a)** Graph representing flow cytometry data, as percentage of positive cells of the following populations: CD45R^+^, CD3^+^, CD11b^+^, GR1^+^, CD11b^+^/GR1^+^, and F4/80^+^, found in bone marrow of healthy WT (n = 5) and Ats1-KO (n = 5) mice. **(b)** Graph representing the mRNA fold change expression of *Cd3g*, *Il12a*, *Cd11b*, *Cd163 and Nos2* genes in bone marrow of healthy WT (n = 5) and Ats1-KO (n = 5) mice, being all values relative to WT. **(c)** Western blot analysis with anti-DPEAAE antibody for ADAMTS-cleaved versican in BM protein extracts from WT and Ats1-KO mice. Blue arrows indicate predicted versican (V0/V1) fragments (around 220 kDa and 70 kDa). Bottom panel includes tubulin staining (grey arrow). Full-length blots are presented in Supplementary Figure 5. **(d)** Graph representing the absolute Ct value for mRNA expression of *Adamts1*, *4*, *5*, *9*, *15*, *and 20* genes in bone marrow of healthy WT (n = 6) and Ats1-KO (n = 5) mice (values higher than 37–38 are considered very low or absent). For all the graphs, results are shown as the median with s.e.m. and statistical significance (*p < 0.05; **p < 0.01; ***p < 0.001; ****p < 0.0001).

We next evaluated the BM of WT and Ats1-KO mice. As in the spleen, our flow cytometry analyses showed no changes in the CD45R^+^ population, and a significant increase in CD3^+^ cells in BM of Ats1-KO mice (Fig. 3a). Despite the low amount of this population in BM, their role has also been highlighted^30^. Furthermore, a remarkable increase of myeloid cells, including CD11b^+^, GR1^+^, and CD11b^+^/GR1^+^, were detected in Ats1*-*KO samples (Fig. 3a), now in clear opposition to our findings in spleen (Fig. 2b). Parallel gene expression analyses corroborated these results. Firstly, the increase in T cells observed by flow cytometry, though not as important as in the spleen, was confirmed by higher levels of *Cd3g*^25^ and *Il12a*^28^ expression in the BM of Ats1-KO mice (Fig. 3b). Secondly, the increase in myeloid cells in the BM of Ats1-KO mice was also supported by the increased gene expression of *Cd11b* (*Itgam*), *Cd163*^31^ and *Nos2*^32^ (Fig. 3b).

The evaluation of VCAN in the BM by immunofluorescence displayed a very faint signal, making putative differences undetectable. Indeed, Western blot analysis showed proteolytic fragments of VCAN in both WT and Ats1-KO samples and the cleavage appeared compromised in the absence of *Adamts1* (Fig. 3c). Finally, gene expression data of ADAMTSs with versicanase activity also confirmed a higher expression of *Adamts5* (Fig. 3d) as previously found in spleen.

In general, these results corroborated previous findings regarding the alteration of organs during adult life in the absence of *Adamts1*^10–13^. However, to our knowledge, this is the first study showing that the lack of ADAMTS1 affects the spleen and BM. The observed variations of the substrate VCAN also supported the overall fluctuations of immune populations.

### The immune infiltration in B16F1 tumours is altered in Ats1-KO mice

Unexpectedly, our results revealed a pro-inflammatory phenotype in the immune organs of the Ats1-KO mice, mainly characterized by an increase in CD3^+^ T cells in the spleen, together with an enrichment of the myeloid fraction in the BM. At this point, we recalled our previous study where we showed that the progression of syngeneic B16F1 tumours was blocked in Ats1-KO mice^9^. In fact, our study highlighted the alteration of immune infiltrates in B16F1 tumours in an ADAMTS1-dependent manner. Our current results encouraged us to perform a deeper study of the immune-related parameters in B16F1 tumour-bearing mice, analysing not only the tumours but also the spleen and BM.

**Figure 4:**
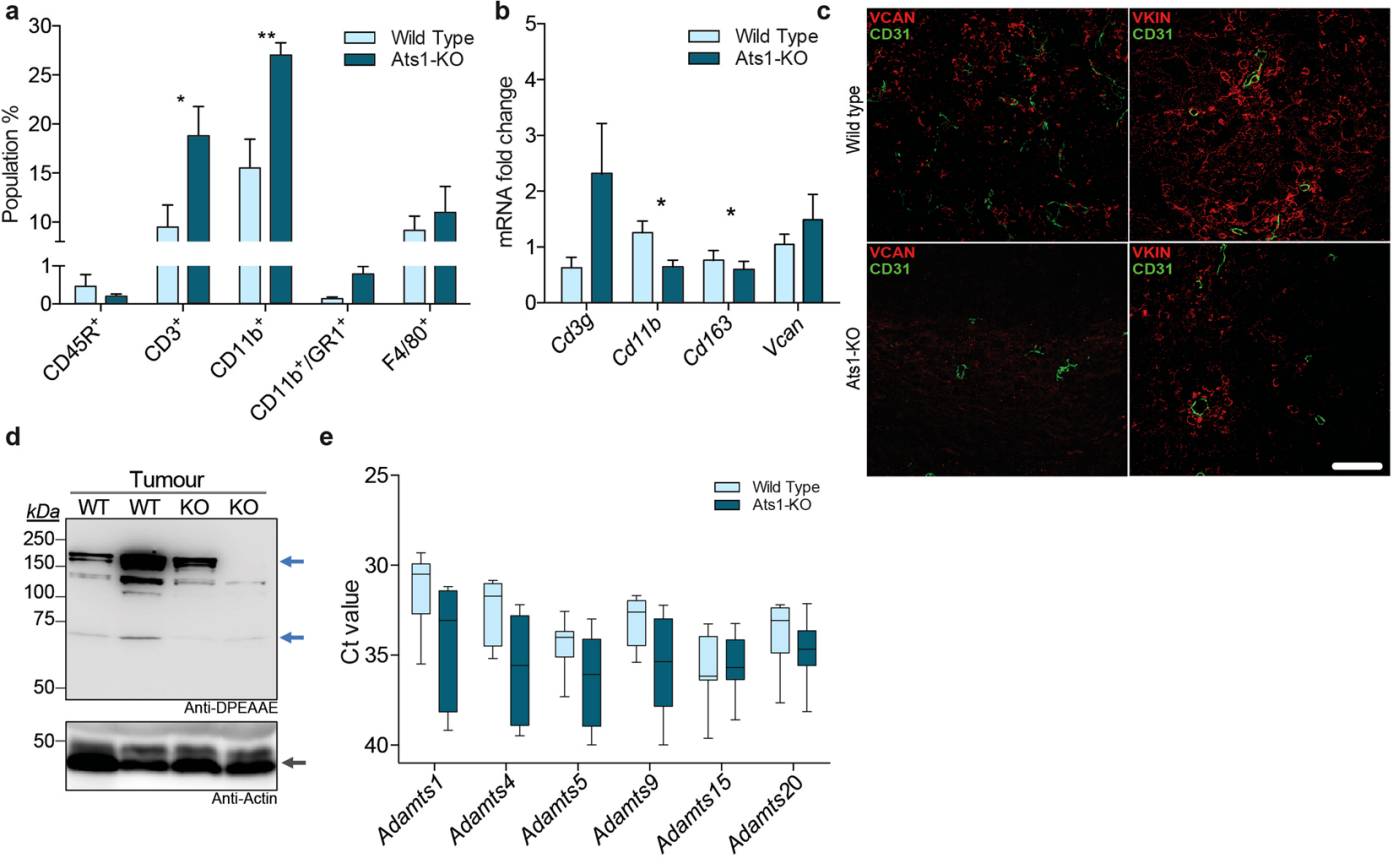
Characterization of immune infiltration, versican and versicanases in B16F1 tumors in WT and Ats1-KO mice. **(a)** Graph representing flow cytometry data, as percentage of positive cells of the following populations: CD45R^+^, CD3^+^, CD11b^+^, CD11b^+^/GR1^+^, and F4/80^+^, found in tumours of WT (n = 5) and Ats1-KO (n = 5) mice. **(b)** Graph representing the relative mRNA fold change expression of *Cd3g*, *Cd11b*, *Cd163* and *Vcan* genes in tumours of WT (n = 5) and Ats1-KO (n = 5) mice. **(c)** Representative images of tumour sections from WT and Ats1-KO mice, showing VCAN and VKIN (red) (left and right column, respectively), and CD31 (green) immunofluorescence staining. Images correspond to a 63x magnification (white scale bar = 20 μm). **(d)** Western blot analysis with anti-DPEAAE antibody for ADAMTS-cleaved versican in tumour protein extracts from WT and Ats1-KO mice. Blue arrows indicate predicted versican (V0/V1) fragments (around 220 kDa and 70 kDa). Bottom panel includes actin staining (grey arrow). Full-length blots are presented in Supplementary Figure 6. **(e)** Graph representing the absolute Ct value for mRNA expression of *Adamts1*, *4*, *5*, *9*, *15*, *and 20* genes in tumours of healthy WT (n = 9) and Ats1-KO (n = 8) mice (values higher than 37–38 are considered very low or absent). For all the graphs, results are shown as the median with s.e.m. and statistical significance (*p < 0.05; **p < 0.01).

First, we addressed the immune infiltration in B16F1 tumours on our different experimental backgrounds using flow cytometry. Infiltration of CD45R^+^ cells appeared near irrelevant without differences between WT and Ats1-KO animals (Fig. 4a). Importantly, we detected a remarkable infiltration of CD3^+^ cells in B16F1 tumours which was significantly higher in Ats1-KO versus WT mice (Fig. 4a), supporting the decreased tumour growth rate in *Adamts1*-deficient animals^9^. The increase in cytotoxic cells was reinforced by induction of gene expression of the related gene *Cd3g* in Ats1-KO tumours (Fig. 4b).

In addition, flow cytometry results showed that CD11b^+^ myeloid cells also increased in Ats1-KO tumours (Fig. 4a). However, the percentage of CD11b^+^/GR1^+^ cells, corresponding to myeloid-derived suppressor cells (MDSCs), was very low (Fig. 4a). Finally, these analyses showed a relevant infiltration of F4/80^+^ cells without alterations between the WT and Ats1-KO groups. Surprisingly, *Cd11b* gene expression data did not match these cytometry results (Fig. 4b). Moreover, *Cd163*, a significant marker of an immunosuppressive scenario^33^, was also significantly downregulated in Ats1-KO tumours (Fig. 4b), also supporting the decreased tumour progression.

Finally, we assessed the presence of VCAN in these tumours. While *Vcan* gene expression did not show significant differences (Fig. 4b), the immunofluorescent detection of full length VCAN revealed an increased deposition in WT versus Ats1-KO tumours and a similar finding was observed for the fragment VKIN (Fig. 4c). In agreement, Western blot also showed an increased presence of proteolysis fragments in WT tumours, although we should highlight the high heterogeneity that we observed between samples (Fig. 4d). As for the overall presence of further ADAMTSs versicanases, we did not find significant differences between WT and Ats1-KO specimens (Fig. 4e).

### Impact of B16F1 tumour growth on spleen and BM of WT and Ats1-KO mice

Our results showing the alteration of immune organs and the modified immune infiltration in tumours encouraged us to evaluate both the spleen and BM in B16F1 tumour-bearing mice.

**Figure 5:**
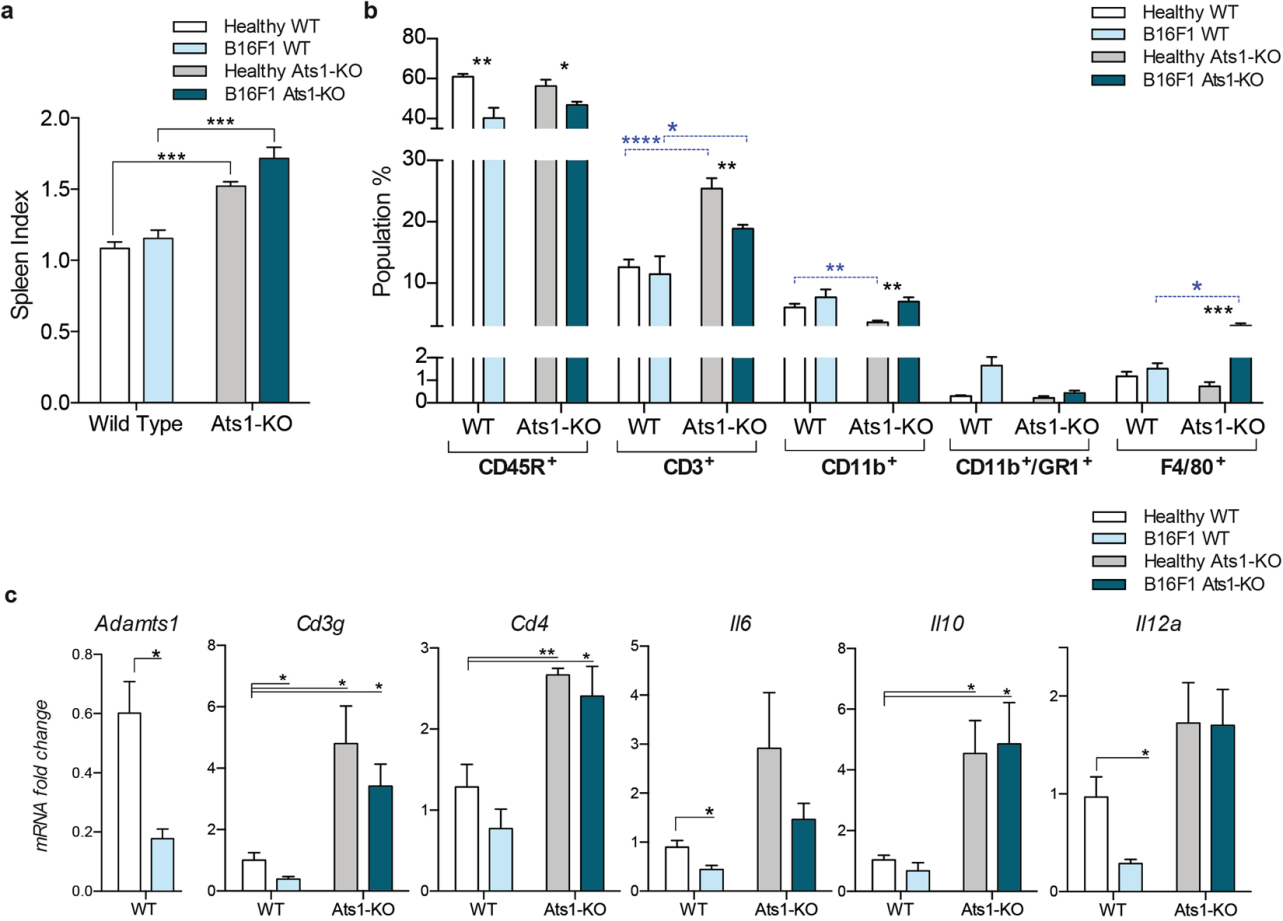
Characterization of spleens from B16F1 tumour-bearing WT and Ats1-KO mice. **(a)** Graph representing spleen index (as indicated in Methods section) of healthy and tumour-bearing mice (WT, n = 6; Ats1-KO, n = 5) to assess splenomegaly. **(b)** Graph representing flow cytometry data, as percentage of positive cells of the following populations: CD45R^+^, CD3^+^, CD11b^+^, CD11b^+^/GR1^+^, and F4/80^+^, found in spleens of healthy WT (n = 6) and tumour-bearing WT (n = 5), and healthy Ats1-KO (n = 5) and tumour-bearing Ats1-KO (n = 5) mice. Blue-dashed bars represent comparison between WT and Ats1-KO groups. **(c)** Graph representing the mRNA fold change expression of *Adamts1*, *Cd3g*, *Cd4*, *Il6*, *Il10*, and *Il12a* in spleens of healthy WT (n = 5) and tumour-bearing WT (n = 5) and healthy Ats1-KO (n = 5) and tumour-bearing Ats1-KO (n = 6) mice. All values are relative to healthy WT. For all the graphs, results are shown as the median with s.e.m. and statistical significance (*p < 0.05; **p < 0.01; ***p < 0.001; ****p < 0.0001).

Regarding the study of spleen, and in line with our previous results, we confirmed the splenomegaly in Ats1-KO mice in the presence of B16F1 tumours, as occurred in healthy mice (Fig. 5a). Flow cytometry analysis showed that the presence of B16F1 tumours reduced the CD45R^+^ population in both WT and Ats1-KO spleens (Fig. 5b). More importantly, and in agreement with our findings in healthy mice, the percentage of CD3^+^ cells was significantly increased in spleens of Ats1*-*KO mice in the presence of tumours, although not as notably as in healthy animals (Fig. 5b). Finally, myeloid-related populations were also affected, though it is important to consider their low levels in this organ. For instance, CD11b^+^ cells increased in spleens of tumour-bearing mice, on both WT and Ats1-KO backgrounds, and F4/80^+^ cells were increased just in the spleens of tumour-bearing Ats1-KO mice. However, no changes were observed in CD11b^+^/GR1^+^ cells (Fig. 5b). In line with our previous results shown in Fig. 2, we completed these studies by evaluating the gene expression of relevant molecules, including *Adamts1* and others implicated in a putative cytotoxic scenario. First, we noticed a decrease of *Adamts1* in spleens of tumour-bearing mice (Fig. 5c), confirming its fine regulation during tumour growth. In relation to the increase in T cells, our results confirmed the induction of genes as *Cd3g*, *Cd4*, *Il6*, *Il10* and *Il12a* in spleens of Ats1-KO mice, independently of the absence or presence of B16F1 tumours (Fig. 5c). These evaluations also showed that the presence of tumours in WT mice provoked a slight but significant decrease in *Cd3g*, *Il6* and *Il12a* expression in this immune organ (Fig. 5c).

**Figure 6:**
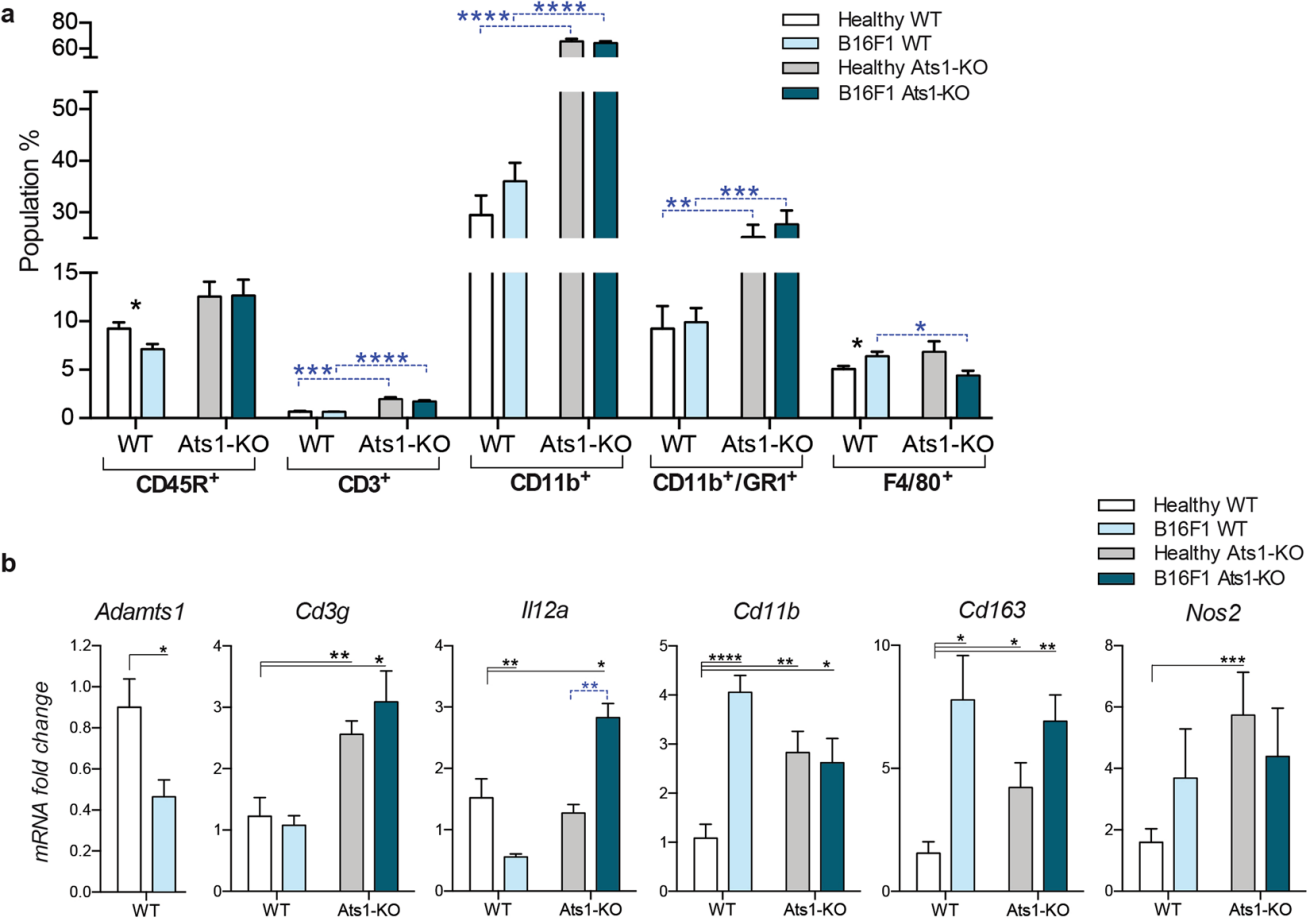
Characterization of bone marrow from B16F1 tumour-bearing WT and Ats1-KO mice. **(a)** Graph representing flow cytometry data, as percentage of positive cells of the following populations: CD45R^+^, CD3^+^, CD11b^+^, CD11b^+^/GR1^+^, and F4/80^+^, found in bone marrow of healthy WT (n = 6) and tumour-bearing WT (n = 6) and healthy Ats1-KO (n = 6) and tumour-bearing Ats1-KO (n = 5) mice. Blue-dashed bars represent comparison between WT and Ats1-KO groups. **(b)** Graph representing the mRNA fold change expression of *Adamts1*, *Cd3g*, *Il12a*, *Cd11b*, *Cd163* and *Nos2* in healthy WT (n = 6) and tumour-bearing WT (n = 5) and healthy Ats1-KO (n = 5) and tumour-bearing Ats1-KO (n = 6) mice. Black bars represent comparison with healthy WT samples. Blue-dashed bar represent comparison between healthy and tumour-bearing Ats1-KO. For all the graphs, results are shown as the median with s.e.m. and statistical significance (*p < 0.05; **p < 0.01; ***p < 0.001; ****p < 0.0001).

We finally proceeded with the study of the BM of our experimental groups. Flow cytometry analysis showed that CD45R^+^ cells were slightly decreased in tumour-bearing WT but not in Ats1-KO animals (Fig. 6a). Interestingly, and in agreement with the spleen, we observed an enhancement of the CD3^+^ population in the BM of both healthy and tumour-bearing WT and Ats1-KO animals (Fig. 6a). Regarding the myeloid populations, which are more relevant in this tissue, we already highlighted the main increase of CD11b^+^ and CD11b^+^/GR1^+^ cells in the BM of healthy Ats1-KO mice, now similarly affected in the presence of tumours (Fig. 6a). Finally, the number of F4/80^+^ cells, which increased in the BM of tumour-bearing WT mice, was significantly reduced on the Ats1-KO background (Fig. 6a). Our parallel gene expression analysis showed a significant downregulation of *Adamts1* in the BM of B16F1-bearing mice (Fig. 6b), as observed in spleens (Fig. 5c). Likewise, immune-related genes such as *Cd3g* and *Il12a* appeared altered in a similar manner on the Ats1-KO background (Fig. 6b), independently of the presence of B16F1 tumours and in line with the increase in T cells observed by flow cytometry. While our previous data on healthy mice corroborated an increase of myeloid markers in the BM of Ats1-KO mice, now we detected that the induction of B16F1 tumours provoked a clear upregulation of genes as *Cd11b* and *Cd163* but just in WT animals (Fig. 6b), without major alteration on the Ats1-KO background, suggesting the blockade of the mobilization of these populations in an *Adamts1*-deficient scenario.

## Discussion

In our attempt to better understand the biological actions of the endogenous extracellular protease ADAMTS1, we performed a new study involving Ats1-KO mice. Although recent investigations are remarking and consolidating the contribution of this protease in the fields of angiogenesis and cancer, the present work unveils a novel and relevant implication of ADAMTS1 in immune-related organs such as spleen and bone marrow, which roles are being highlighted by recent advances in immuno-oncology^34,35^.

**Figure 7:**
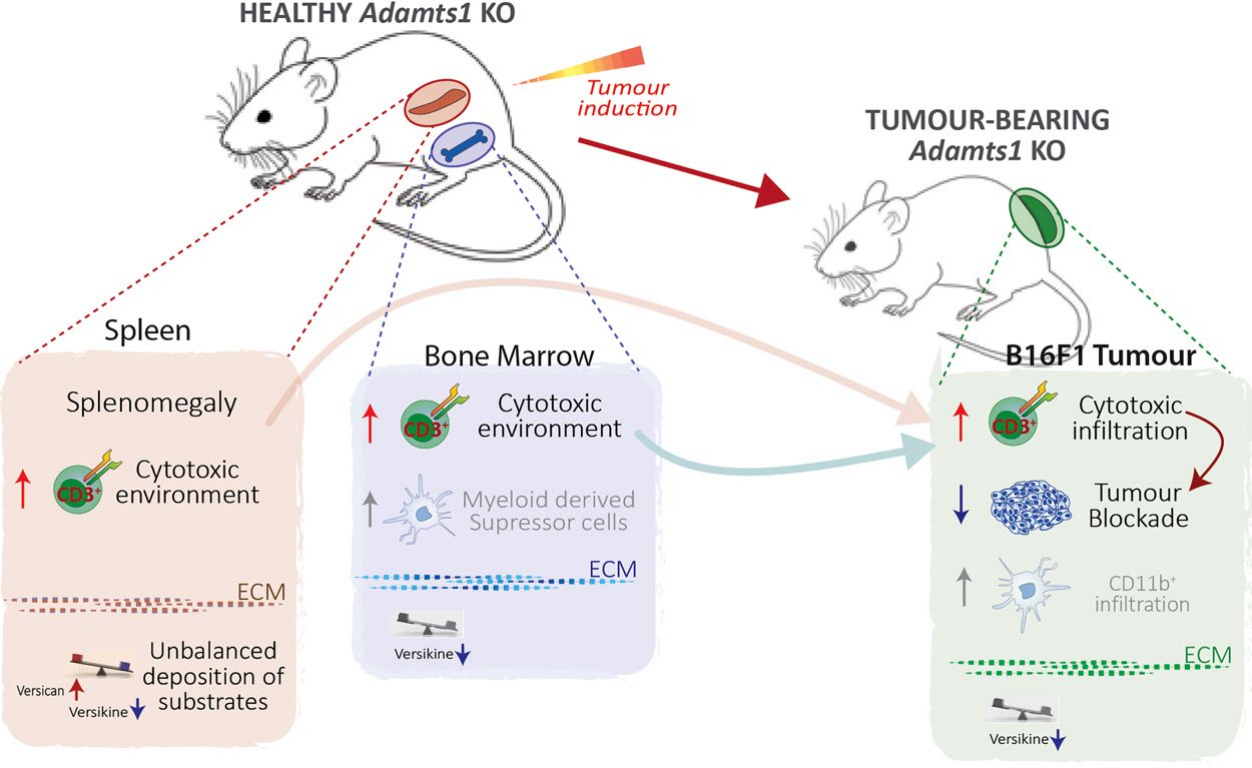
Scheme representing the observed alteration of immune organs in *Adamts1* KO mice with consequences for tumour progression. Healthy Ats1-KO displayed an unbalance of specific immune populations in spleen and bone marrow, remarking the increase of cytotoxic cells in both organs. Our present study also shows a different deposition of the relevant ADAMTS1 substrate versican in the ECM of spleen and BM, suggesting their regulatory role. As reported, B16F1 tumour progression is significantly blocked in Ats1-KO mice and the analysis of such tumours revealed an increased infiltration of CD3+ cells, in full agreement with the detected alterations in immune organs, and also correlating with the altered levels of versican. Results in both healthy and tumour-bearing mice supported the role of ADAMTS1 as a modulator of the immune cell response by the alteration of its substrates, with relevant consequences for final tumour progression.

Driven by our interest in the activity of ADAMTS proteases on the vasculature^7,9^, our initial gene expression and immunohistological studies showed specific alterations in organs of Ats1-KO mice, like kidney, aorta, and heart, in accordance to the recognized roles of ADAMTS1 during the development and adult life^11–13^. To our surprise, we detected variations on two immune organs, the BM and spleen, which is striking considering the low levels of *Adamts1* expression in both locations. Our initial gene expression results showed alterations of *Nid1* and *Vcan* in both BM and spleen, probably representing a consequence of the alteration of these organs since earlier developmental stages. Indeed, a deeper study confirmed our suspicion that the immune system was disturbed in *Adamts1*-deficient animals (Fig. 7).

We found that *Adamts1* deficiency led to a pro-inflammatory setting in the spleen, mostly showing a relevant increase in the number of CD3^+^ cells, correlating with the obvious splenomegaly of these animals. Furthermore, the BM displayed a more complex landscape. T cells were also increased in the BM of Ats1-KO mice but it was accompanied by a clear induction of myeloid populations such as CD11b^+^, GR1^+^, and CD11b^+^/GR1^+^ cells. Motivated by the recognized action of ADAMTS1 on the vasculature, our closer evaluation of both organs did not show clear effects in terms of the vessel density, although a slight decrease of the vessel perimeter was detected in the BM, deserving further investigations. Moreover, the immunostaining of VCAN and Nid1 revealed interesting and motivating results. To date, studies of the implication of Nid1 in the immune system are very limited but the importance of the basement membrane has been remarked^36^. For VCAN, recent reports suggested that its cleavage by ADAMTS proteases can induce a general immunogenic response in the tumour microenvironment^37,38^. Our analyses identified alterations of VCAN in the spleen and the BM (Fig. 7), although we remark that proteolysis is still occurring even in the absence of *Adamts1* due to the activity of other versicanases. We need to consider that these organs have been impacted by the absence of *Adamts1* during development so its alterations observed at adult life are the result of such modified scenario.

Certainly, our findings seem relevant in the context of tumour growth (Fig. 7). Our published results with the B16F1 model already determined a clear impairment of tumour progression in the absence of stromal ADAMTS1^9^. In addition to the identified changes in the tumour vasculature, we have now revealed the increased infiltration of CD3^+^ T cells in B16F1 tumours in the *Adamts1*-deficient environment, encountering in this case a scenario with a general decrease of VCAN and VKIN, though the role of additional substrates is likewise expected. The origin of these immune cells is still unknown, but our data confirms that both spleen and BM of Ats1-KO mice showed a parallel and significant increase of this population, in healthy but also in tumour-bearing animals. Certainly the BM has received a major attention in the literature, although predominantly focused on the contribution of myeloid cells for tumour progression^39^. The role of the spleen has being emphasized for tumour-induced immune tolerance^35^, but the changes that this organ suffers during tumour progression have not been as deeply assessed. Significantly, a recent report shows how the different locations of a tumour triggered distinct immune responses in the spleen^40^, and the nature of the tumour can also provoke important differences^39,41^ as we have shown here.

We also need to mention the important increase of CD11b^+^ cells in the tumours in the *Adamts1*-deficient context. Indeed, this population seems to be the more abundant within the immune infiltrate in our model. This result is apparently contradictory with the blockade of tumour growth although the controversy is currently present in the scientific community. Paradoxically our gene expression data show an opposite finding for *Cd11b* itself, but also for significant MDSC markers, such as *Cd163*, observations that need to be taken into account for further research. Surely it is necessary to remark the low levels of MDSC CD11b^+^/GR1^+^ cells and the lack of differences for F4/80^+^ cells in the tumour niche, also reported for its pro-tumorigenic role^42^. Accordingly, we considered that these cells are not enough to compete with the CD3^+^ T-lymphocyte infiltration. Our findings suggest a complex communication between the tumour and distinct organs, in line with the discovery of educational mechanisms of distant immune organs, such as the involvement of exosomes^43^, contributing to the final fate of the tumour.

To date, the knowledge of the functional relationships of ADAMTS proteases with the immune system is very limited and undefined. Importantly, a recent report showed a regulatory role of ADAMTS5 for influenza-specific T cell immunity involving the substrate VCAN^44^, and additional works support the contribution of VCAN proteolysis influencing the balance of immune populations in tumours^37,38^. Significantly, the expression of versican has been associated to both pro- and anti-tumorigenic scenarios^45,46^, so the regulation of its proteolysis, in conjunction with the nature of further constituents in the microenvironment, surely are key features to explore. In this setting, the recent work by Asano *et al*.^45^ shows a remarkable colocalization of the fragment VKIN with endothelial cells in the tumour vasculature suggesting a leading role modulating angiogenesis. As we did not observe such colocalization in our experimental approaches, this fact reflects once more the relevance of tumour heterogeneity among different models and supports the need to keep investigating the ECM within a tumour.

Further works highlight relationships between ADAMTSs and inflammatory processes, such as ADAMTS4 and ischemic stroke^47^, or ADAMTS1 itself and the development of aortic aneurysms^13^. Another interesting work reported significant levels of ADAMTS1 in interstitial fluid of spleens, lymph nodes and plasma after a systemic lipopolysaccharide-induced inflammation^48^. At this point, we should recall the original cloning of mouse *Adamts1* in a cachexia model^6^, a phenomena with a recognized inflammatory component. However, no further studies have focused exactly on this context, excluding a few references that revealed its genetic regulation by a variety of cytokines^6,49^.

In conclusion, we have described here a promising and still little explored pro-inflammatory landscape, correlating with a halt of B16F1 tumours in *Adamts1*-deficient mice. Our results in both healthy and tumour-bearing mice supports the role of ADAMTS1 as a modulator of the immune cell response inspiring additional efforts to investigate its transcriptional regulation, the interaction with some of its more relevant substrates, and also its relationship with pathways involving cytotoxic immune responses. The nature of this protease reflects a new scenario where the mutual regulation between vasculature and immune infiltration needs to be outlined^50^.

## Methods

### Mouse colony handling

C57BL/6 wild type and Ats1-KO mice^51^ were maintained and bred at the *Centro de Investigación Biomédica-UGR* animal facility. Mice were properly housed on a 12 h day/night cycle in sterilised cages, under pathogen-free conditions, and provided food and water *ad libitum*. Mice were sex and age matched for different experiments. For genotyping, genomic DNA was isolated from ear samples using the Nucleospin tissue kit (Macherey-Nagel). To evaluate splenomegaly, spleen index was determined according to the formula: Spleen index = square root of spleen weight (x100) divided by body weight^52^. All mouse experiments were performed in accordance with relevant guidelines and regulations, approved by UGR ethical committee for animal research (Number 152-CEEA-OH-2016).

### Cell culture and tumour growth

B16F1 murine melanoma cells were cultured in DMEM supplemented with 10% fetal calf serum (FCS) and 1% of penicillin/streptomycin under standard conditions (37 °C, 5% CO_2_ and 95% relative humidity).

For the generation of syngeneic tumours, 1 × 10^6^ B16F1 cells in 100 μl of Phosphate-buffered saline (PBS), were subcutaneously injected in the right flank of WT and Ats1-KO C57Bl/6 mice. Animal weight and tumour size were monitored every 3 days after cell injection. These assays were performed up to 21 days or until the tumour reached 1 cm in length. Tumour dimensions were measured with a digital calliper. Final tumour volume was calculated *ex vivo* according to the formula: tumour volume = (π × length × width × height)/6. All animals were sacrificed following proper ethical guidelines, and tumours were dissected and processed for further analysis, as indicated below.

### *Ex vivo* T cell proliferation assay

Spleens from C57BL/6 WT and Ats1-KO mice were homogenized and red blood cells were lysed by resuspending and incubating the samples in Ammonium-Chloride-Potassium (ACK) buffer pH 7.2 for 4 min at room temperature. Splenocytes (50 × 10^6^ cells/staining) were subsequently labelled with 2 µM of carboxyfluorescein diacetate succinimidyl ester (CFSE) for 5 minutes at room temperature^24^. To reduce CFSE toxicity, 2 ml of heat-inactivated serum were added and the cells were washed and counted using trypan blue. CFSE-labelled splenocytes were seeded in a flat-bottom 96 well plate (0.2 × 10^6^ cells/well) and were stimulated with anti-CD3 Ab (553058, BD Bioscience) at 2 µg/ml. For the negative control without stimulation, complete RPMI was added. After 3–4 days, cells were harvested and stained for flow cytometry according to described protocols. Antibodies used for flow cytometry were: rat anti-mouse CD4-APC (17-0041-81, ThermoFisher) and rat anti-mouse CD8a-APC-Cy^TM^7 (557654, BD Biosciences).

### Quantitative RT-PCR

Total RNA was extracted from tissues and tumour biopsies using the NucleoSpin RNAII kit (Macherery-Nagel). cDNA was synthesized with iScript cDNA Synthesis Kit (BioRad). qPCR reactions were performed in a 7900HT PCR machine (Applied Biosystems) using the Fast SYBR green master mix (Applied Biosystems). qPCR representations show the 2^(ΔΔCt)^ value (indicated as mRNA fold change in all the graphs), using the 18S gene as a housekeeping control. Values show median ± standard error of the mean (s.e.m.). Primers used for these assays are indicated in Supplementary Table 1.

### Immunohistochemistry

For the morphometric analysis of vasculature, sections from spleens and bone marrow from WT and Ats1-KO mice were subjected to immunofluorescence staining with a monoclonal rat anti-mouse Endomucin antibody (SC-65495, SCBT). Images were captured with the AxioImager A1 microscope (Zeiss), and converted to binary for further analysis with Image J software as indicated^9^. For additional immunofluorescence determinations, sections were incubated with the following antibodies: polyclonal rabbit CD31 (AB28364, Abcam), polyclonal goat Nid1 (AF2570, R&D), polyclonal rabbit anti-mouse Versican V1 (2701534, Millipore), polyclonal rabbit anti-mouse Versican DPE (Versikine) (Ab19345, Abcam). Fluorescence confocal images were captured with a LSM 710 confocal microscope (Zeiss).

### Western blot analysis

Total protein from tumour samples and cell lysates was extracted using an extraction buffer containing: 6 M Urea, 50 mM Sodium acetate, 0,1% triton, 1 mM EDTA, 1 mM PMSF, 1 mM bestatin, 1 mM pepstatin, and 1 mM aprotinin. For VCAN analyses, all samples were incubated 1 h at 37 °C with Chondroitinase ABC (C3667, Sigma-Aldrich), in chondroitinase buffer (180 mM Tris, 216 mM Sodium Acetate) with Trypsin inhibitor (from chicken egg white, T9253, Sigma-Aldrich). Proteins were resolved by SDS-PAGE and transferred to Polyvinylidene difluoride (PVDF) membranes (BioRad). Membranes were blocked with 5% low-fat milk and incubated with the polyclonal rabbit anti-Versican DPE antibody (ab19345, Abcam) that recognizes ADAMTS-1/4 cleavage site, and monoclonal mouse anti-Actin (sc-8432, Santa Cruz Biotechnology) and monoclonal rat anti-alpha Tubulin (sc-53029, Santa Cruz Biotechnology), as loading controls. After incubation with the appropriate secondary peroxidase-conjugated antibody, signal was detected with the Amersham ECL Prime Western Blotting Detection Reagent (GE Healthcare Life Sciences) in an ImageQuant LAS4000 (GE Healthcare Life Sciences).

### Flow Cytometry

To obtain a cell suspension, every type of sample is processed differently as follows: spleens were disrupted physically using a 5 ml syringe as a pestle; bone marrow was flushed out the tibia of the animals with PBS; and B16F1 tumours required a previous mechanical disaggregation process with scissors. At this point, all samples were filtered throughout a 70 μm cell strainer and cell suspensions were incubated with collagenase 0, 5% (C2799, Sigma-Aldrich) in PBS for 1 hour at 37 °C, shaking from time to time to avoid clump formation. After incubation, cell suspension was flushed through a 19.5 G needle, added to DMEM supplemented with 10% serum, and spun 5 min at 300 g. All the samples were suspended in Ammonium-Chloride-Potassium (ACK) buffer pH 7.2 and incubated during 4 min at room temperature, to remove red blood cells. Finally, cells were spun and pellet was resuspended in FACS buffer (PBS 1×, 1% FCS and 2 mM EDTA) for cell counting and antibody incubation.

To identify dead cells, we added blocking antibody (2.4G2 anti-mouse CD16/CD32–553142, BD Bioscience) in a 1% BSA, 1% FCS and 7- Amino-Actinomycin D (7-AAD) blocking solution and incubated for 5 min at room temperature. Then, conjugated-primary antibody solution was added and incubated for 30 min at room temperature. After incubation, cells were spun at 300 g during 5 min and finally resuspended in FACS buffer. Flow cytometry analyses have been performed using the FACS Canto II and DIVA software (BD Bioscience). The process of gating and population selection is detailed in Supplementary Fig. 2. Antibodies used for flow cytometry were: rat anti-mouse CD11b-APC (553312, BD Bioscience), rat anti-mouse F4/80-PE (123110, Biolegend), rat anti-mouse/human CD45R/B220-FITC (110452, Thermofisher), hamster monoclonal anti-mouse CD3e-PE (12-0031, Thermofisher), rat anti-mouse GR-1-FITC (RB6-8C5, Miltenyi Biotech).

### Statistical analysis

All statistical analyses were performed using GraphPad Prism (GraphPad software Inc.). For quantitative RT-PCR, flow cytometry analyses, and spleen index, Student’s t test was performed in paired groups of samples with known median. Error bars show the standard error of mean (s.e.m.). Previously, studies of the median and outliers were performed in all the sample cohorts by Tukey’s test.

## Electronic supplementary material

Supplementary Information

## Data Availability

All data generated or analysed during this study are included in this published article (and its Supplementary Information files).
