# Supplementary material for: ADAMTS1 protease is required for a balanced immune cell repertoire and tumour inflammatory response
Source: Sci Rep. 2018 Aug 30;8:13103. doi: 10.1038/s41598-018-31288-7 (PMC6117274; doi:10.1038/s41598-018-31288-7)
Supplement: Supplementary file 1 — Supplementary Information [file 41598_2018_31288_MOESM1_ESM.pdf]

Supporting Information

ADAMTS1 protease is required for a balanced immune cell repertoire and tumour inflammatory response

Francisco Javier Rodríguez-Baena**, Silvia Redondo-García**, Carlos Peris-Torres, Estefanía Martino-Echarri, Rubén Fernández-Rodríguez, María del Carmen Plaza-Calonge, Per Anderson, and Juan Carlos Rodríguez-Manzaneque*

Supplementary Figure 1. Gene expression of vasculature-related genes and characterization of vasculature of spleen and bone marrow of WT and Ats1-KO mice.

(a) Graphs representing the mRNA fold change expression of vasculature-related genes *Pecam1*, *Cspg4* and *Acta2* in organs from WT (n=5) and Ats1-KO (n=5) mice. All values are relative to WT. **(b and c)** Representative binary fluorescent images of spleen (b) and bone marrow (c) sections showing Endomucin staining used for vasculature analysis (black scale bar = 200 µm). Adjacent graphs represent vessel density (VD) (vessel number/mm^2) and average vessel perimeter (VP) (µm) of spleens (b) from WT (n=5) and Ats1-KO (n=5) mice and bone marrow (c) from WT (n=3) and Ats1-KO (n=2) mice. All results are shown as the median with s.e.m. and statistical significance (*, $p < 0.05$; **, $p < 0.01$; ***, p < 0,001).

While spleen did not show any changes between WT and Ats1-KO mice in parameters such as vessel density (VD) and vessel perimeter (VP), the evaluation of BM samples showed a decreased vessel parameter in Ats1-KO mice that deserve future analyses.

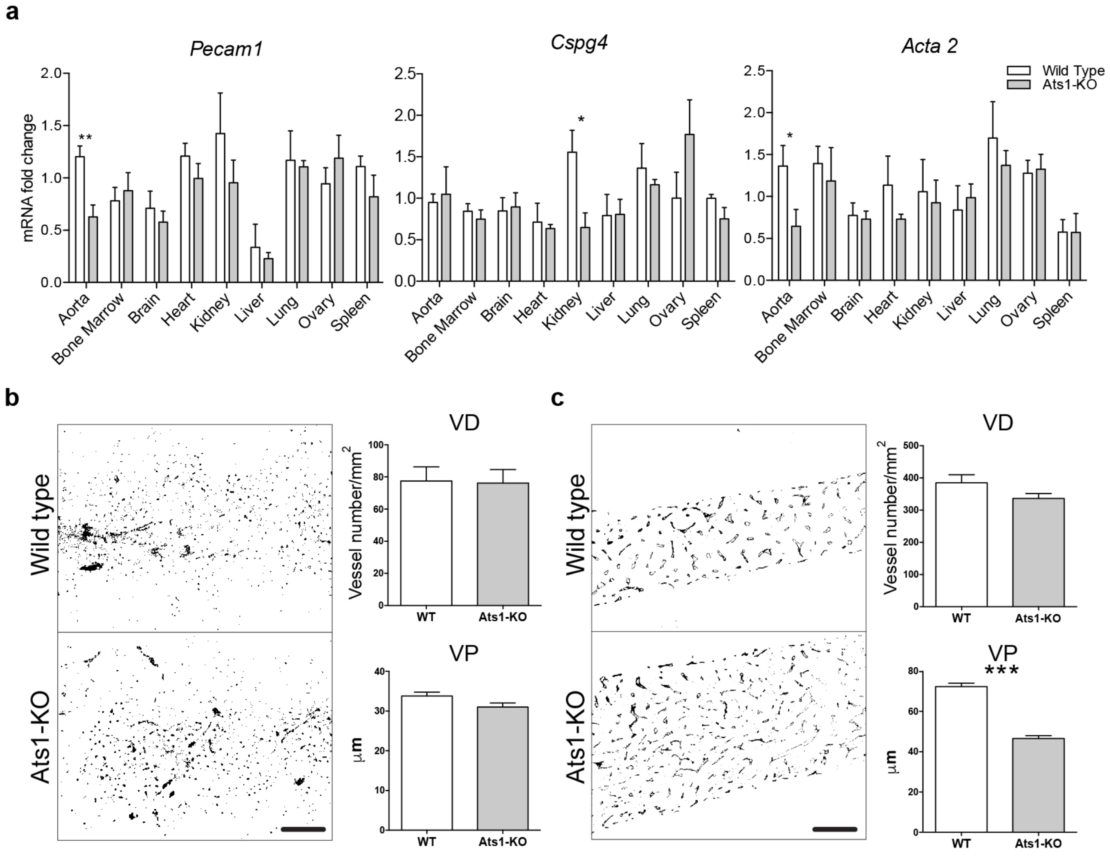

Supplementary Figure 2. Analysis of spleens of WT and Ats1-KO mice.

(a) Hematoxilin and Eosin (H&E) staining of spleen sections from WT and Ats1-KO mice. **(b)** Graphs representing proliferation of CD4 and CD8 cell populations previously isolated from whole fresh spleens of WT and Ats1-KO mice. These experiments were performed according to recognized CFSE assay (reference 24) detailed in the Methods section. The represented values correspond to: initial cells prior to stimulation, non-labelled; 4 days culture without stimulus; 3 days culture with stimulus; and 4 days culture with stimulus.

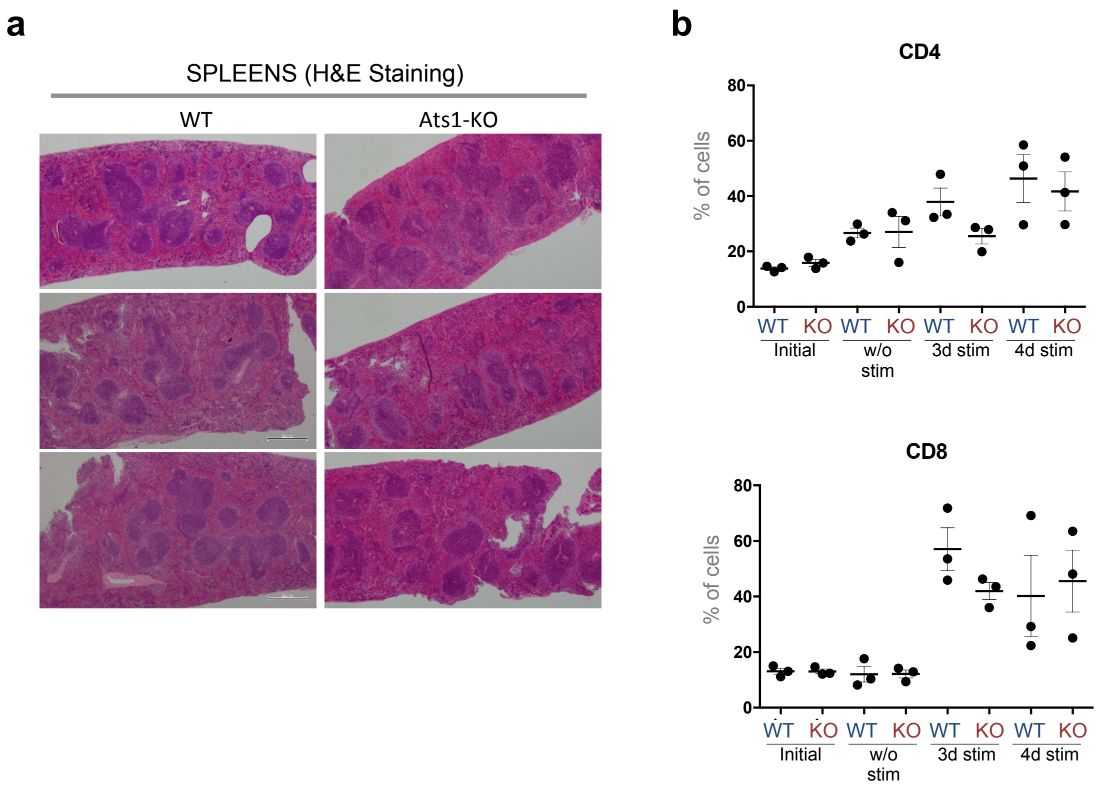

Supplementary Figure 3. Gating and population selection for the representation of flow cytometry data.

The following panels represent the populations that have been selected during our flow cytometry analyses. Although the process has been the same, we showed an example of spleen (a) and bone marrow (b) of healthy WT mice, and a B16F1 tumor (c). First, the main population was gated to avoid cell debris and doublets. Following, negative 7-AAD cells (using the PerCP channel) were selected as living population. An equal number of these living cells were incubated with two antibody combinations as follows: i) CD11b-APC Ab, CD45R-FITC Ab and CD3-PE Ab; and ii) CD11b-APC Ab, F4/80-PE Ab and GR1-FITC Ab. The resulting density blots for each antibody combination have been gathered in grey-dashed boxes. Those populations that have been represented in graphs throughout the manuscript are named in black ($CD45R^+$, $CD3^+$, $CD11b^+$, $GR1^+$, $CD11b^+/GR1^+$, and $F4/80^+$), together with its specific percentage data for each example. The populations named in grey have not been selected for representation. Same parameters were applied to every sample and medians were obtained from every group in order to be finally represented.

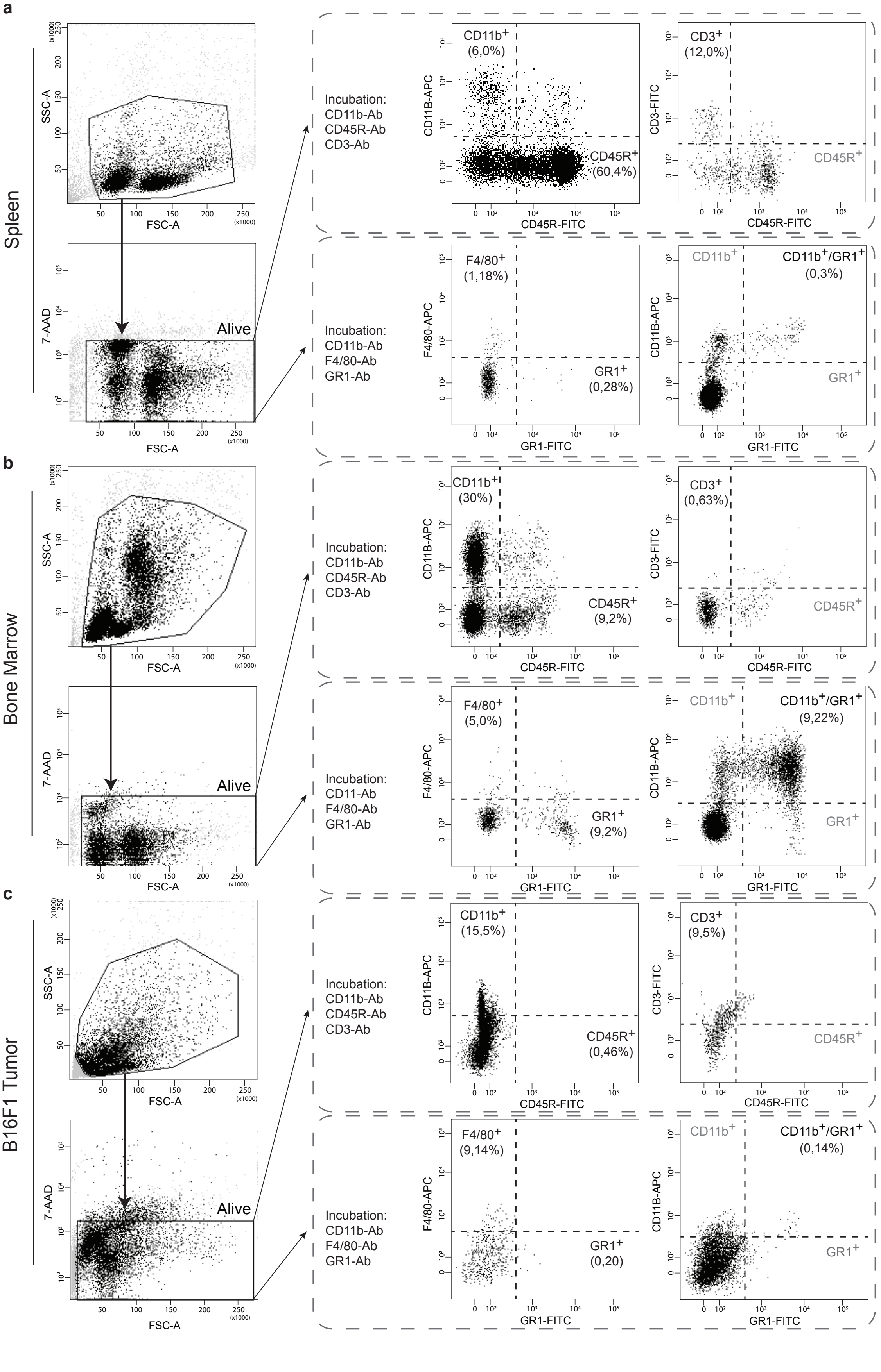

a
Spleen
SSC-A
FSC-A
7-AAD
Alive
Incubation:
CD11b-Ab
CD45R-Ab
CD3-Ab
CD11b⁺ (6,0%)
CD45R⁺ (60,4%)
CD11B-APC
CD45R-FITC
CD3⁺ (12,0%)
CD3-FITC
CD45R⁺
Incubation:
CD11b-Ab
F4/80-Ab
GR1-Ab
F4/80⁺ (1,18%)
GR1⁺ (0,28%)
F4/80-APC
GR1-FITC
CD11b⁺
CD11b⁺/GR1⁺ (0,3%)
GR1⁺
b
Bone Marrow
CD11b⁺ (30%)
CD45R⁺ (9,2%)
CD3⁺ (0,63%)
Incubation:
CD11-Ab
F4/80-Ab
GR1-Ab
F4/80⁺ (5,0%)
GR1⁺ (9,2%)
CD11b⁺/GR1⁺ (9,22%)
c
B16F1 Tumor
CD11b⁺ (15,5%)
CD45R⁺ (0,46%)
CD3⁺ (9,5%)
F4/80⁺ (9,14%)
GR1⁺ (0,20)
CD11b⁺/GR1⁺ (0,14%)

Supplementary Figure 4. Full-length blots of Figure 2e.

Red square represents the part of the image showed in Figure 2e.

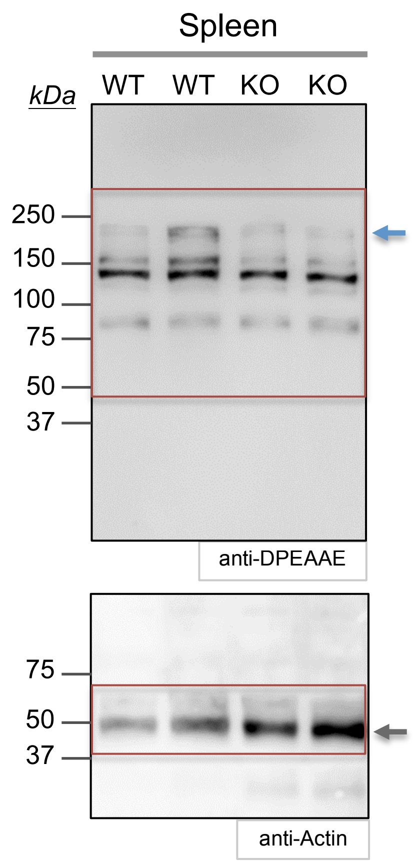

Supplementary Figure 5. Full-length blots of Figure 3c.

Red square represents the part of the image showed in Figure 3c.

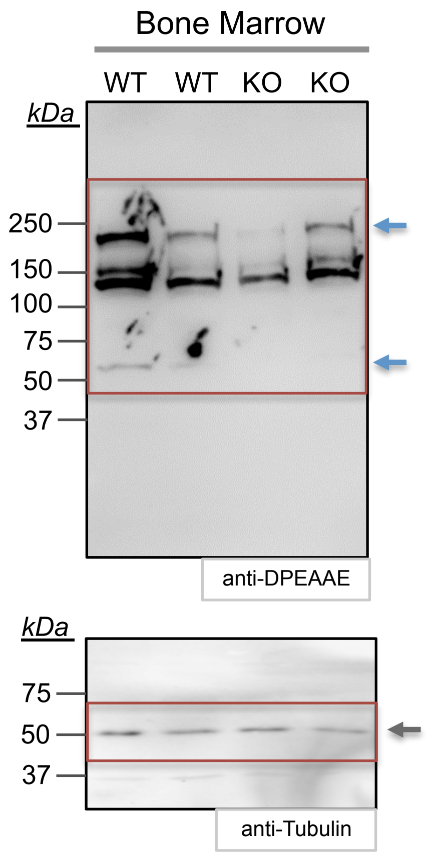

Supplementary Figure 6. Full-length blots of Figure 4d.

Red square represents the part of the image showed in Figure 4d.

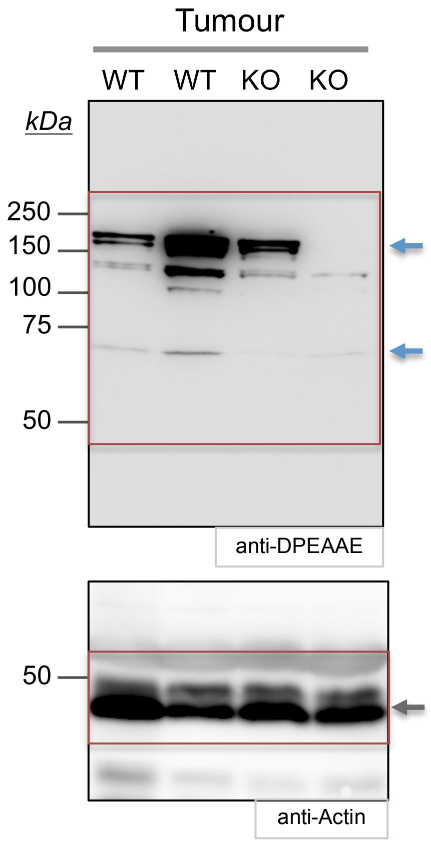

Supplementary Table 1. List of primers used for quantitative RT-PCR.

Gene Name	Forward sequence	Reverse sequence
18s	CAAATCGCTCCACCAACTAA	GATGACCACTTCGGACATTATGAG
Acta2	TCTTTCATTGGGATGGAGTCAG	GACAGGACGTTGTTAGCATAGA
Adamts1	CTGGCAGAAACAACACAACAG	TGAATTGGGCCATGTGTTTAAC
Adamts15	TGATCTGTCTCCGACCCTCA	GACTCACCATGCCCACT
Adamts20	AATGTGCCAAGGTCTTCATAGA	AAACAGTGGCATCGGTAAGT
Adamts4	CAGACGAAGCACTCACCTT	CCAGCCTGAGGAACATTGA
Adamts5	AAATGGCAGCACCAACATAAC	TGATGCCCACATAAATCCTCTC
Adamts9	GCCTGTGCTACCTTACCTAAAC	CCACAAGTCACGGAACAAGAG
Cd11b	GCAGCACTGAGATCCTGTTTA	CTCCACTTTGGTCTCTGTCTT
Cd163	ACGGCACTCTTGGTTTGT	GAGGAAACTGTAAGTCGCTGAA
Cd3g	CAGTCAAGAGCTTCAGACAAG	GATGGCTGTACTGGTCATATTC
Cd4	GAGTTCCCAGAAGAAGATCAC	AAGGCGAACCTCCTCTAA
Cspg4	GACGGCGCACACACTTCTC	CAGACTCTGGACAGACGGTCAA
Foxp3	CAATAGTTCCTTCCCAGAGTTC	TCGGATAAGGGTGGCATAG
Interleukin 10	TGTCAAATTCATTCATGGCCT	ATCGATTTCTCCCCTGTGA A
Interleukin 12a	CTGGAACTACACAAGAACGAGAG	GGCACAGGGTCATCATCAAA
Interleukin 6	TAGTCCTTCCTACCCCAATTTCC	TTGGTCCTTAGCCACTCCTTC
Nidogen 1	CGGTCTATGTCACCACAAATGGTA	AGGTTCCGGGATGGTATTCTGT
Nidogen 2	TTCCTGTCCCTCCTCTGGAA	GCCATTATACGTGAAGACTTGATCAT
Nos2	CTTGGTGAAAGTGGTGTTCTTTG	TCAGACTTCCCTGTCTCAGTAG
Pecam1	ACCCTTCTGCTCTGTTCAAG	GGGTCAGGTTCTTCCCATTT
Versican	CTTTGCTCATCGACGCACA	TGTCATTGAGGCCGATCCA
